# Is the injection of tramadol effective at control of pain after impacted mandibular third molar extractions? A systematic review and meta-analysis

**DOI:** 10.4317/medoral.25498

**Published:** 2022-08-17

**Authors:** Kalyne Kelly Negromonte Gonçalves, Marcelo Soares dos Santos, Davi da Silva Barbirato, Caio César Gonçalves Silva, Allan Vinícius Martins de Barros, Emerllyn Shayane Martins de Araújo, Renata de Albuquerque Cavalcanti Almeida, Belmiro Cavalcanti do Egito Vasconcelos

**Affiliations:** 1 PhD student in Oral and Maxillofacial Surgery. University of Pernambuco, Recife–PE, Brazil; 2 Postdoctoral fellow in Oral and Maxillofacial Surgery. University of Pernambuco, Recife–PE, Brazil; 3 PhD student in Oral Medicine and Pathology. University of Pernambuco, Recife-PE, Brazil; 4 Resident in Oral and Maxillofacial Surgery. Oswaldo Cruz University Hospital, University of Pernambuco, Recife–PE, Brazil; 5 PhD in Oral and Maxillofacial Surgery. University of Pernambuco, Recife–PE, Brazil; 6 Coordinator of the Doctorate and Master’s degree in Oral and Maxillofacial Surgery at University of Pernambuco, Recife–PE, Brazil. Coordinator of the residency in Oral and Maxillofacial Surgery at Hospital da Restauração, Recife–PE, Brazil

## Abstract

**Background:**

Third molar extraction is among the most common surgical procedures performed by oral-maxillofacial surgeons. Postoperative pain, swelling and trismus are common, especially in wisdom teeth, due to trauma to local tissues and the duration of the surgical procedure, among other factors.

**Material and Methods:**

This systematic review was conducted in accordance with the ‘Preferred Reporting Items for Systematic Reviews and Meta-Analyses’ in order to answer the focused question: ‘Is the local submucosal injection of tramadol effective at the control of postoperative pain in patients submitted to impacted mandibular third molar extractions?’. We analyzed papers published until March 30, 2021 in the MEDLINE|PubMed, Web of Science and Cochrane Library databases. Gray literature was also consulted. Standard pairwise meta-analyses of direct comparisons were performed using a fixed-effect model; I2 ≥ 50 % or ≥ 75 % indicated moderate or high heterogeneity, respectively. Risk of bias was assessed by Cochrane Collaboration’s tool.

**Results:**

In total, 172 participants (98 males and 74 females, aged 18 or over) from three randomized placebo-controlled trials were considered for analysis. The submucosal injection of 2 ml of tramadol adjacent to the impacted mandibular third molar was effective in controlling pain up to 6-hours after surgery, in increasing the onset of consumption of rescue analgesic and in reducing the total number of rescue analgesics used.

**Conclusions:**

The submucosal injection of tramadol can be considered a safe and effective procedure for pain control after impacted mandibular third molar extractions.

** Key words:**Tramadol, impacted teeth, third molars, postoperative pain, adverse effects.

## Introduction

Third molar extraction is among the most common surgical procedures performed by oral-maxillofacial surgeons (1,2). Postoperative pain, swelling and trismus are common, especially in wisdom teeth, due to trauma to local tissues and the duration of the surgical procedure, among other factors (3,4).

After the extraction of third molar teeth, monotherapies or combined therapies of analgesics and/or non-steroidal anti-inflammatories are used for pain control. Despite the benefits of these drugs, many patients do not tolerate their use due to adverse effects, such as gastric or duodenal ulcers, platelet disorders, renal failure and bronchospasm (5,6). Moreover, caution is required regarding the use of these drugs on pregnant women, nursing mothers, diabetics, immunosuppressed individuals and those with vascular diseases (1). The therapeutic individualization strategy for analgesic and anti-inflammatory drugs usage and their route of administration must occurs (7,8).

Opiates are a viable medicinal alternative for the control of moderate to intense acute pain following third molar extraction (9). Tramadol [(1R,2R)-2-[(dimethylamino)methyl]-1-(3-methoxyphenyl) cyclohexan-1-ol)] is classified as a weak, central action opiate that is clinically effective for the control of moderate to intense pain (10). It is an agonist of the μ-opioid receptor that reduces the transmission of pain impulses by inhibiting the reuptake of serotonin and norepinephrine (11,12).

Tramadol can be administered through enteral (oral and rectal) or parenteral (intravenous, intramuscular and submucosal) routes. The local submucosal injection of tramadol after third molar extraction has been studied due to the proximity to the surgical site and low systemic absorption; moreover, this procedure is simple to perform and there is a low frequency of adverse effects (13).

The efficacy, posology and route of administration of tramadol after third molar extractions have not yet been established, which compromise the decision-making process in clinical practice. Therefore, this systematic review aims to answer the focal question developed in accordance with the recognized Patient, Intervention, Comparison, and Outcome (PICO) format: ‘Is the local submucosal injection of tramadol effective at the control of postoperative pain in patients submitted to impacted mandibular third molar extractions?’.

## Material and Methods

- Protocol and registration

This systematic review was conducted in compliance with the ‘Preferred Reporting Items for Systematic Reviews and Meta-Analyses’ (PRISMA) (14,15). PROSPERO registration protocol #CRD42020150445.

- Literature search strategy

Searches were performed in the MEDLINE|PubMed, Web of Science and Cochrane Library databases for articles published up to March 30, 2021, using MeSH terms and other free terms, combined by the Boolean operators "OR" and "AND": “tramadol AND third molar” OR “tramadol AND impacted tooth” OR “tramadol AND tooth extraction” OR “analgesics, opioid AND third molar” OR “analgesics, opioid AND impacted tooth” OR “analgesics, opioid AND tooth extraction” OR “tramadol hydrochloride AND third molar” OR “tramadol hydrochloride AND impacted tooth” OR “tramadol hydrochloride AND tooth extraction” OR “opioids AND third molar” OR “opioids AND impacted tooth” OR “opioids AND tooth extraction”. The gray literature was accessed by consulting the Brazilian Digital Library of Theses and Dissertations (BDTD) and www.ensaiosclinicos.gov.br databases. Hand-searches were also performed in specialized periodicals (British Journal of Oral and Maxillofacial Surgery; International Journal of Oral and Maxillofacial Surgery; Journal of Dentistry; Medicine and Medical Sciences; Journal of Cranio-Maxillo-Facial Surgery; Journal of Oral and Maxillofacial Surgery; and Oral Surgery, Oral Medicine, Oral Pathology, Oral Radiology), and in reference lists of selected articles.

- Selection and eligibility criteria

The controlled vocabulary (MeSH terms) and free keywords in the search strategy were defined to identify clinical trials based on the elements of the PICO question:

1) Participants (P) = patients undergoing impacted mandibular third molar extraction

2) Intervention (I) = use of local submucosal injection of tramadol

3) Comparison (C) = placebo, oral tramadol, intravenous tramadol, or intramuscular tramadol

4) Outcomes (O) = postoperative pain

Secondary outcomes: use of rescue analgesics and adverse effects.

Inclusion criteria: i- randomized controlled trials; ii- studies in which intervention group received local submucosal injection of tramadol, and the control group received a placebo, oral tramadol, intravenous tramadol or intramuscular tramadol out of the operation site; iii- studies that evaluated postoperative pain using a subjective measure, such as the visual analog scale; and iv- studies published in English, Spanish or Portuguese. Exclusion criteria: i- studies that did not evaluate the primary or secondary outcomes of interest; ii- lack of information on dose or administration route of tramadol; iii- studies that used systemic tramadol (e.g., intramuscular) or other analgesics or anti-inflammatories in the pre or postoperative period (except rescue analgesics), in addition to submucous injection of tramadol adjacent to the third molar; iv- pain assessment presented only in the form of graphs and not provided by the authors; v- studies not related to the subject; and vi- inability to access the full text. No time or language restrictions was applied.

The selection process was conducted in two phases: Phase 1, two researchers (K.K.N.G. and M.S.S.) independently examined the titles and abstracts of all identified references, applying the including process (blind process); and Phase 2, the same two reviewers independently applied the exclusion criteria to the other studies, based on reading the full text (blind process). Inter-reviewer reliability in the study selection process was determined by the Cohen κ test, assuming an accepTable threshold value of 0.80 (16). The disagreement at any stage was resolved by discussion and mutual decision (consensus meeting) with a third reviewer (B.C.E.V.). The final decision/selection was always based on the full text of the publication.

- Data extraction

The full texts were evaluated and judged in the entire document. Authors were contacted when necessary to obtain details on study design and data clarification. Data were extracted by two independent reviewers (K.K.N.G. and M.S.S.) from the included studies and described in the paper at a consensus meeting with the third reviewer.

- Summary measures and synthesis of the results

Qualitative data were analyzed and presented in the form of text and Tables. Standard pairwise meta-analyses of direct comparisons were performed using a fixed-effect model, and results were expressed as mean difference and relative 95 % CI (confidence interval). Heterogeneity was assessed by using the Chi-square-based Q-statistic method and Higgins inconsistency measurement (I2), with significance indicated by *P* ≤ 0.05. The I² test ≥ 50 % indicates moderate heterogeneity and values ≥ 75 % indicate high heterogeneity (17-19). The effect estimate was calculated through the mean differences, adopting the statistical method of inverse variance.

- Risk of bias

Within studies: Once a detailed appraisal of the methods and results was performed independently by two researchers (K.K.N.G. and M.S.S.), the studies were analyzed to determine the possibility of biased results, using 'Cochrane Collaboration’s Tool for assessing risk of bias in randomized trials' from seven domains: random sequence generation, allocation concealment, blinding of participants and personnel, blinding of outcome assessment, incomplete outcome data, selective reporting, and other bias (20,21).

Across studies: The presence of publication bias was investigated for the outcome of interest based on visual detection/analysis of the funnel plot (22,23), using the RevMan 5.4 software (Review Manager, version 5.4, Nordic Cochrane Centre, Cochrane Collaboration, September 2020).

## Results

- Study selection

**Figure 1:**
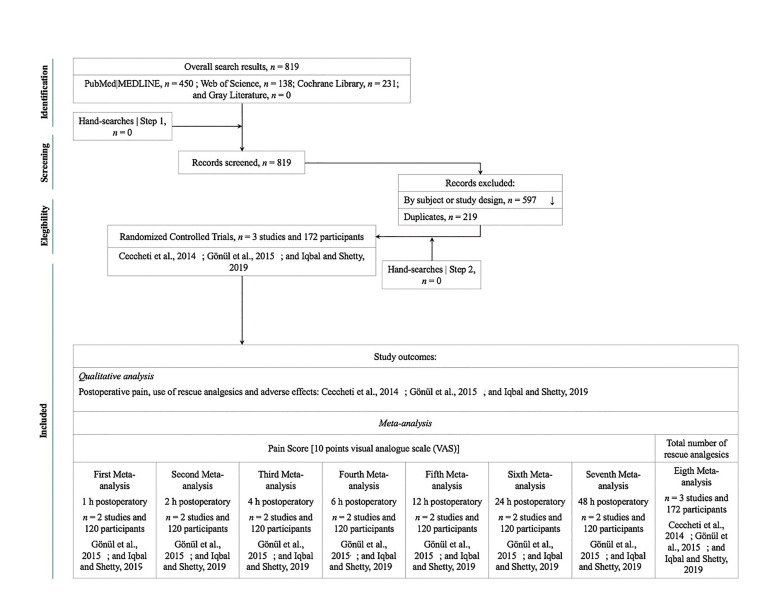
Screening and enrolment. Legend: PRISMA flow diagram showing selection of articles for overview; n, absolute frequency.

The searches of the databases led to the retrieval of 819 records (MEDLINE|PubMed), n = 450; Web of Science, n = 138; and Cochrane Library, n = 231). The studies were imported to the reference manager (EndNote Online), which led to the removal of 219 duplicates. The analysis of the titles and abstracts, followed by the full text, resulted in the exclusion of 597 papers (Fig. 1). Inter-evaluator agreement in this step was calculated using kappa correlation coefficient (MEDLINE|PubMed, = 0.92; Web of Science, = 1.0; and Cochrane Library, = 0.89), confirming a high level of agreement between the reviewers. No additional publications were found in the gray literature, the specialized periodicals or reference lists of the studies included.

- Characteristics of Included Studies

In total, 172 participants (98 males and 74 females, aged 18 or over) from three randomized placebo-controlled trials were considered for analysis (10,24,25). The submucosal injection of 2 ml of tramadol adjacent to the impacted mandibular third molar was administered at doses of 1 mg/kg, 50 mg and 100 mg, after surgery. The placebo group received a submucosal injection of 2 ml of sterile saline solution injection. Postoperative pain was assessed between 0.5 hours and 72 hours after surgery, using the pain score [10 points visual analogue scale (VAS)]. Local anesthetics and rescue analgesic drugs differed between studies (Table 1).

- Risk of Bias

For the quality assessment, the articles were classified according to the seven domains of risk of bias (20,21): random sequence generation, allocation concealment and selective reporting showed low risk of bias, while the blinding of participants and personnel and blinding of outcome assessment were not clear, representing the main risk of bias. There were incomplete outcome data in the result section of studies, and there was no standardization in the classification of third molars (other risks of bias), both interpreted as uncertain risk of bias (Supplement 1).

- Synthesis of the Results

**Figure 2:**
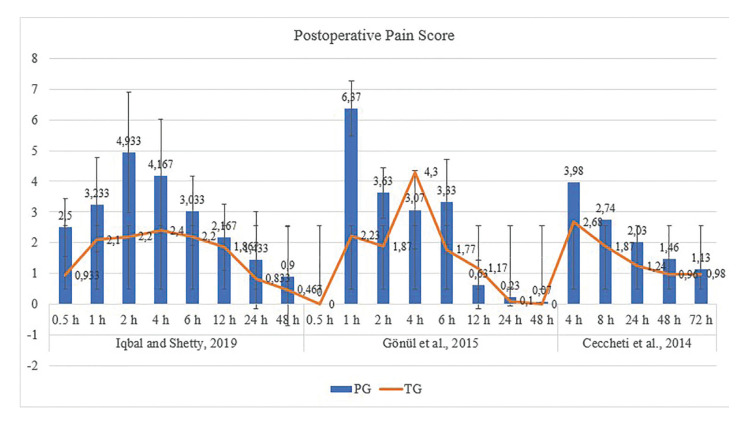
Clustered column graph of mean pain score in the postoperative period. Legend: PG, placebo group; and TG, tramadol group. Data: mean and standard deviation values.

Iqbal *et al*. (2019) (10) reported the lowest postoperative pain scores and onset of consumption of rescue analgesic, highest number of rescue analgesics used. Ceccheti *et al*. (2014) (24) used tramadol 100 mg, followed by 1 mg/kg and 50 mg used by Gönül *et al*. (2015) (25) and Iqbal *et al*. (2019) (10), respectively. At all doses of tramadol, the postoperative pain score, onset of consumption of rescue analgesic and total number of rescue analgesics were significantly lower in the tramadol group (TG), compared with the placebo group (PG) (Fig. 2, Supplement 2). No adverse effects were seen in TG 50 mg. Nausea, vomiting, dizziness and burning were reported in the other two doses of tramadol (1 mg/kg and 100 mg) (Table 2).

**Table 1:**
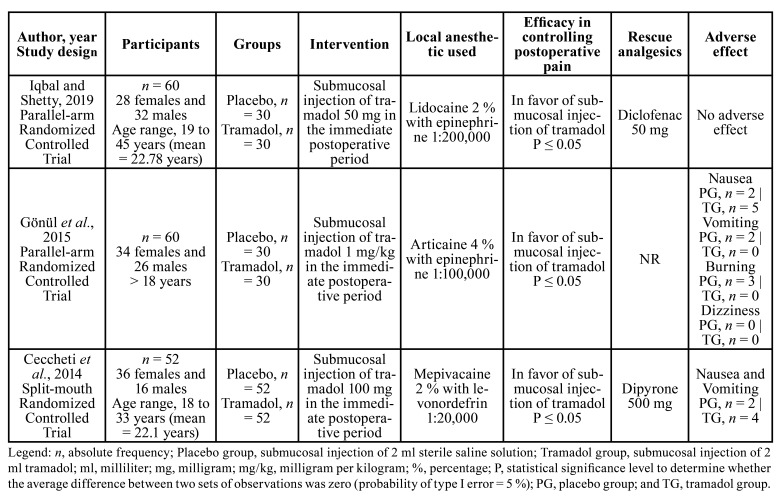
Descriptive data on study design and qualitative results.

**Table 2:**
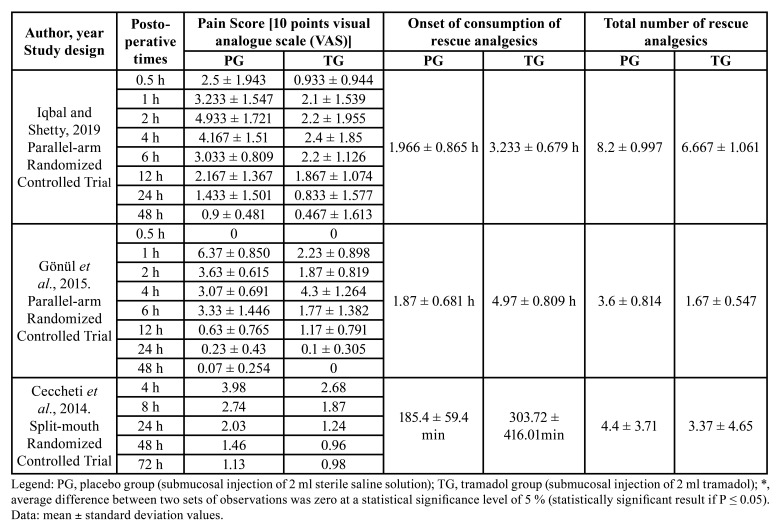
Quantitative data on outcomes of interest.

- Meta-analysis for postoperative pain

**Figure 3:**
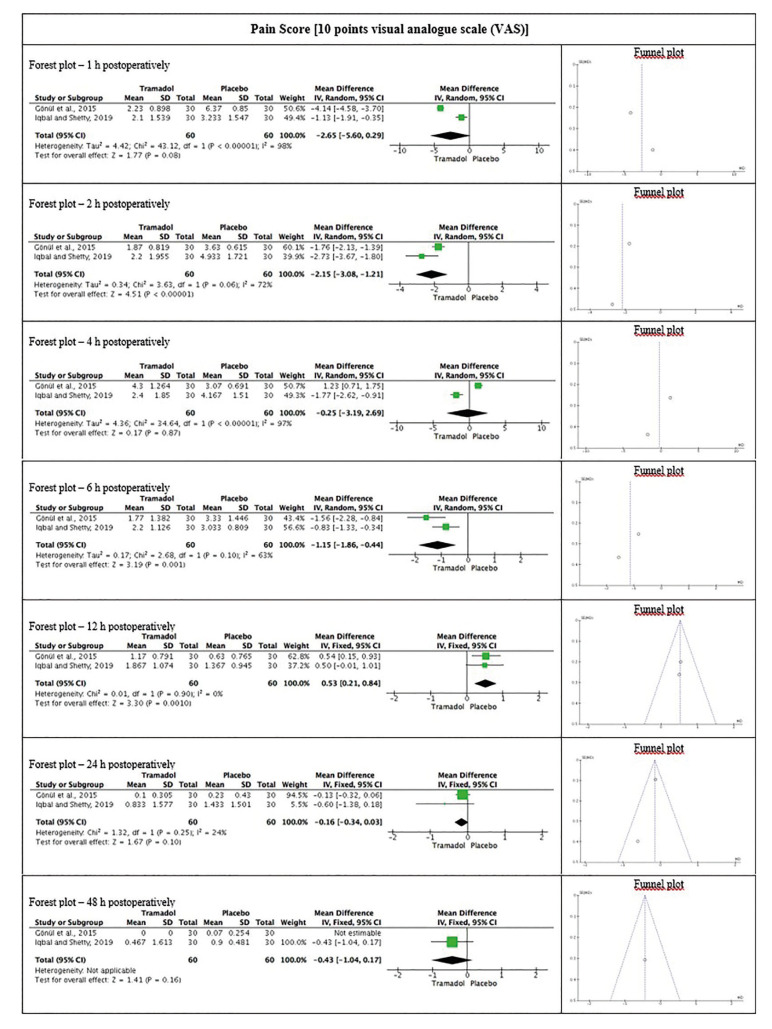
Meta-analysis on the efficacy of submucosal injection of tramadol for postoperative pain after third molar extraction, compared to placebo group.

Submucosal injection of tramadol significantly reduced pain 2-hours [2, *P* > 0.05; I2 = 72 %; mean difference -2.15 (95 % CI = -3.08 to -1.21)] and 6-hours after surgery [2, *P* > 0.05; I2 = 63 %; mean difference -1.15 (95 % CI = -1.86 to -0.44)]. Although not statistically significant, the 1-hour, 24-hours and 48-hours postoperative meta-analyzes suggest better pain control in favor of tramadol (Fig. 3).

**Figure 4:**
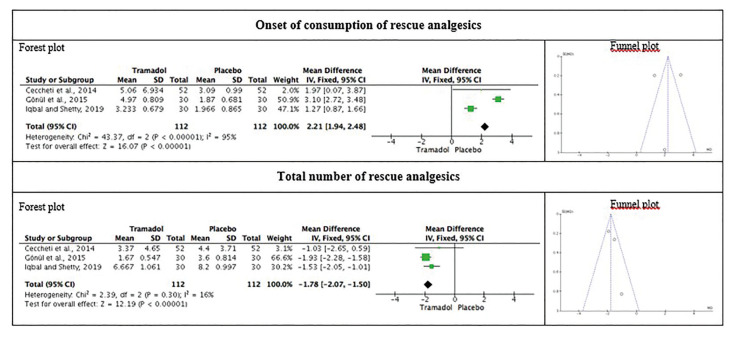
Meta-analysis on the ‘Onset of consumption of rescue analgesics’ and ‘Total number of rescue analgesics’ used after third molar extraction, comparing the submucosal injection of tramadol with the placebo group.

The total number of rescue analgesics consumed was significantly lower in TG [2, *P* > 0.05; I2 = 16 %; mean difference -1.78 (95 % CI = -2.07 to -1.50)], compared to PG (Fig. 4).

## Discussion

The submucosal injection of 2 ml of tramadol adjacent to the impacted mandibular third molar was effective in controlling pain up to 6-hours after surgery, with benefits observed up to 48-hours postoperatively. Consequently, the total number of rescue analgesics used was significantly lower in the TG compared to the PG. In addition, the onset of consumption of rescue analgesic in the TG was approximately twice that observed in the PG.

Tramadol is an atypical opioid analgesic, with opioidergic, noradrenergic and serotonergic actions, well tolerated and effective in controlling moderate pain due to its opioidergic and monoaminergic activities. Tramadol modulates the cellular response to pain through voltage-gated sodium ion channels, V1 channels of transient receptor potential, glutamate receptors, α2 adrenoceptor receptors, adenosine receptors and mechanisms involving the substance P, related peptide to the calcitonin gene, prostaglandin E2 and pro-inflammatory cytokines downregulation. It also modifies the crosstalk between neuronal and non-neuronal cells in peripheral and central tissues and modulates neuronal hyperexcitability in these regions. Due to the wide spectrum of molecular targets, tramadol monotherapy relieves several types of pain, such as post-operative, lumbar and neuropathic, and is associated with childbirth, osteoarthritis, fibromyalgia and cancer, being used as a well-tolerated alternative to other drugs (26,27).

Like lidocaine, tramadol's blocking activity not only suppresses nerve conduction, but also inhibits ectopic activities in sensitized neurons. Both have high affinity to fast inactivated sodium ion channels than to resting channels and exhibit usage-dependent blockage. Tramadol 50 mg leads to membrane-stabilizing with a higher local concentration (IC50 = 21 μM) than in plasma (1 μM) (27,28). According to Pozos-Guillén *et al*. (2005) (29), tramadol prolongs the anesthetic effect and postoperative analgesic effectiveness, prolonging the onset of the need for rescue analgesics and reducing the number of these drugs in the postoperative period. Then, the postoperative benefit of submucosal injection of tramadol immediately after impacted mandibular third molar extraction can be perceived by the patient as a significant extension of local anesthesia.

Furthermore, tramadol it acts on opioid receptors and seems to modify the transmission of pain, inhibiting the reuptake of monoamines. It has been described that µ-opioid receptor agonists act to inhibit activation of adenylyl cyclase and tetrodotoxin-resistant Na+ channels on peripheral afferent neurons produced by inflammatory mediators such as prostaglandin E2 and serotonin. There is also evidence pointing that opioids inhibit release of substance *P* and calcitonin gene-related peptide from primary afferent neurons, and open ATP-sensitive K+ channels via Gi proteins resulting in hyperpolarization, reduction in firing of the primary afferent neuron and antinociception. Action mechanism of tramadol blocks noradrenaline uptake with selectivity, serotonin uptake, nonspecific voltage-dependent K+ channels and the nitrergic system. It has been demonstrated that tramadol not only inhibits 5-HT reuptake, but also induces 5-HT release in the raphe dorsal nucleus. However, explanations for the local action of tramadol remain unclear. One possible explanation is that the local effect of tramadol is mediated locally in peripheral nerve fibers (30).

Tramadol 50–100 mg intramuscularly showed an effective and well-tolerated postoperative analgesic effect, comparable to morphine, pentazocine and ketorolac. Although tramadol 75 mg was also associated with sedation, adverse effects such as nausea and vomiting were more frequent (26). Even at the injecTable dose of 50 mg of tramadol, burning sensation, pain and local pre-anesthetic erythema may occur (27).

Oral non-steroidal anti-inflammatory drugs are the most used to control postoperative pain in teeth extractions, although they are not always effective alone (28). The combined therapy of ibuprofen 400 mg and oxycodone hydrochloride 5 mg produced the best postoperative analgesia for impacted mandibular third molar extractions. However, adverse effects such as nausea, dizziness and headache have been associated with this therapy (31). The same adverse effects have been reported in two studies included in this systematic review (24,25); Iqbal and Shetty (2019) (10) used the lowest dose of submucosal tramadol (50 mg) and reported no adverse effects among the participants, even with the highest consumption of rescue analgesics among the studies analyzed. However, the tolerability of patients to submucosal injection of tramadol was similar or less than PG, confirming the low occurrence of adverse effects to tramadol (24,25), and suggesting a nocebo effect.

According to Agrawal *et al*. (2019) (32), therapeutic doses of tramadol do not cause the depression of the circulatory or respiratory systems, being considered even safer with local application.

Beyond third molar surgery, local tramadol injections were reported by Demiraran *et al*. (2006) (33) and Alemanno *et al*. (2012) (34), for inguinal herniotomy and arthroscopic surgery for rotator cuff tears, respectively. Local tramadol injection prolonged the duration of anesthesia and reduced the requirement for total analgesics after surgery. These results were also observed in the submucosal injection for impacted mandibular third molar extractions (10,24,25).

The present meta-analysis demonstrated that the submucosal injection of tramadol achieved superior results with statistical significance in terms of pain control compared to the placebo six hours after third molar surgery. This finding may be explained by the fact that the plasma half-life of tramadol is six hours, independently of the administration route, demonstrating that this drug has a beneficial effect, especially with regards to early pain control.

In contrast, the placebo was superior to submucosal tramadol regarding pain control 12 hours after surgery, likely due to the fact that the effect of tramadol had been reduced by half, losing its analgesic effect over time. Moreover, the local application of the drug was progressively absorbed by systemic circulation. At 24 hours, no statistically significant difference was found between the two groups, offering no evidence of a late effect of submucosal injected tramadol.

The three randomized placebo-controlled studies evaluated showed mild methodological heterogeneity, and statistical heterogeneity in the 1-hour and 4-hours postoperative pain score meta-analyzes. The summary measures of pain 2-hours and 6-hours after surgery represented a significant difference of approximately two points in the 10-points VAS, in favor of TG. Descriptive data suggest at least twice as much pain in the PG compared to the TG, for almost all postoperative follow-up.

Some limitations must be considered when interpreting our findings. We believe that the number of clinical trials analyzed and the lack of standardization regarding the classification method for third molar impaction, the degree of surgical difficulty, the duration of surgery and the experience of the surgeon limit the evidence for clinical decision making. There was also no consensus among the studies included in this review regarding the local anesthetic and dose of tramadol used. Besides that, the risk of bias assessment revealed the need for further studies with more precise and clearer methodologies; many domains of the 'Cochrane Collaboration’s Tool for assessing risk of bias in randomized trials' have not been clearly described in the papers.

Despite these limitations, the three randomized placebo-controlled trials included in this systematic review represent suggest the clinical efficacy of local submucosal injection of tramadol to control postoperative pain and reduce consumption of other drugs after surgery, in impacted mandibular third molar extractions.

In conclusion, results of the present systematic review and meta-analysis revealed that submucosal injection of tramadol adjacent to the impacted mandibular third molar is a safe procedure with important analgesics potential, especially in the first 6-hours after surgery. Reducing the demand for rescue analgesics or combination therapies represents an additional benefit of submucosal tramadol for patients. However, caution should be exercised when interpreting these results due to the heterogeneity among studies. We strongly recommend that new RCTs be performed using well-defined methodologies to improve the quality of evidence regarding this topic.
